# Functionalized Gelatin Electrospun Nanofibrous Membranes in Food Packaging: Modification Strategies for Fulfilling Evolving Functional Requirements

**DOI:** 10.3390/polym17081066

**Published:** 2025-04-15

**Authors:** Shiyi Liang, Jian Zhang, Shunfen Huang, Xingzi Lan, Wenlong Wang, Yadong Tang

**Affiliations:** 1 School of Biomedical and Pharmaceutical Sciences, Guangdong University of Technology, Guangzhou 510006, China; 2 State Key Laboratory of Precision Electronic Manufacturing Technology and Equipment, School of Electromechanical Engineering, Guangdong University of Technology, Guangzhou 510006, China; 3 School of Mechanical and Electrical Engineering, Guangzhou University, Guangzhou 510006, China

**Keywords:** electrospinning, gelatin, food packaging, nanofiber, modification

## Abstract

Gelatin, known for its excellent biocompatibility, strong aggregative properties, and low cost, has been extensively investigated as a promising material for food packaging. Among various fabrication methods, electrospinning stands out due to its simplicity, cost-effectiveness, high process controllability, and ability to produce nanofiber membranes with enhanced properties. This review provides a comprehensive overview of the sources, properties, and applications of gelatin, along with the fundamental principles of electrospinning and its applications in food packaging. Additionally, the common types of electrospinning techniques used in food packaging are also covered. In recent years, increasing research efforts have focused on gelatin-based electrospun nanofiber membranes for food packaging applications. The functionalization of electrospinning gelatin-based nanofiber membrane was realized by incorporating various active substances or combining it with other techniques, fulfilling the new requirements of food packaging. In this review, gelatin-based electrospun nanofiber membranes for food packaging applications are overviewed, with a particular emphasis on various types of modifications for the membranes aimed at meeting diverse application demands. Finally, the future perspectives and challenges in the research of gelatin-based electrospun nanofiber membranes for food packaging are discussed.

## 1. Introduction

As a pivotal safeguard in ensuring food safety, food packaging plays a crucial role in shielding food from external environmental factors throughout distribution, thereby extending its shelf life [1]. However, with the deepening emphasis on food safety and the evolving landscape of market demands, the requirements for food packaging are progressively undergoing change and development.

Plastics, owing to their lightweight nature, robust mechanical performance, ease of molding, and various other properties, have found extensive application for food packaging. However, their non-biodegradable nature poses a significant environmental challenge [2]. Aligned with principles of environmental protection and sustainable development, edible/degradable food packaging has emerged as a burgeoning trend [3]. Materials for degradable food packaging are diverse, including plant-derived sources such as grains, legume flours, and cellulose, as well as animal-based materials including marine algae, chitosan, and gelatin [4]. In particular, gelatin has gained extensive attention in biodegradable food packaging due to its biodegradability, good polymerization, and cost-effectiveness [5,6]. Despite these advantages, single gelatin films encounter limitations, such as inadequate waterproofing, poor thermal stability, and low mechanical strength. To overcome these drawbacks, various compounding and modification strategies have been explored, involving the incorporation of functional materials or active agents to enhance the performance of gelatin-based composite films [5,6].

**Figure 1:**
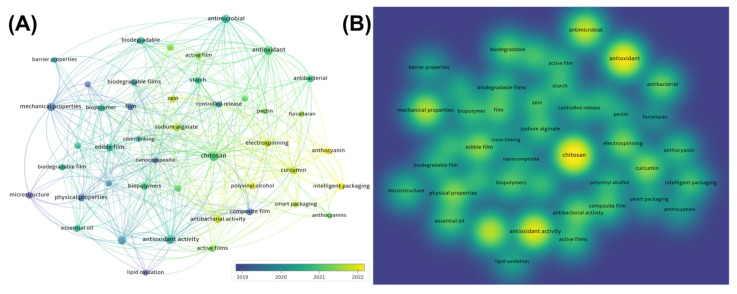
(**A**) A time distribution of different keywords, labeled to represent the year in which the keywords are predominantly concentrated. (**B**) A density analysis of different keywords; the brighter colors indicate more frequent appearances of the keywords. Supported by VOSviewer 1.6.20.

Gelatin-based composite films can be prepared using various methods, such as casting, coatings, electrospinning, and others [7]. Keyword analysis of the literature on the application of gelatin in food packaging published over the years was performed using VOSviewer 1.6.20 software (science citation index and social science citation index from Web of Science database, topic: “(gelatin) and (food packaging)”; additional criteria: exclusion of reviews literature; search date: 14 April 2024). In total, there were 1507 valid publications. Out of a total of 2880 keywords, the keywords with less than 16 repetitions were screened out, leaving 44 keywords. As shown in Figure 1, electrospinning showed a relatively obvious density in the density visual analysis (Figure 1B), and in recent years, electrospinning has become an increasingly popular manufacturing method in the field of gelatin food packaging (Figure 1A). Notably, electrospinning technology offers several advantages: (1) simple and cost-effective manufacturing process; (2) accessible and easily adjustable operating conditions; (3) nanofiber membranes with high surface area, high porosity, and a modifiable surface (high surface area, high porosity, and easily modifiable surface of nanofiber membranes); and (4) high process controllability. Benefiting from these advantages, electrospun nanofibers are highly favored in the realm of food packaging applications [8,9]. Moreover, modified gelatin-based electrospun nanofiber membranes retain the inherent biocompatibility of gelatin while exhibiting enhanced properties such as hydrophobicity, antibacterial activity, and antioxidant capacity, making them an ideal candidate for functional food packaging.

Previous research has provided reviews on electrospun nanofibers in the context of food packaging [10,11,12,13], as well as on gelatin in food packaging [5,7,14]. However, a dedicated review focusing on electrospun gelatin-based materials in food packaging remains absent. This article aims to fill this gap by providing a thorough review of electrospun gelatin-based nanofiber membranes for food packaging applications. It primarily elucidates the fundamental concepts of gelatin and electrospinning, while emphasizing the exploration of various modification methods of electrospun gelatin nanofiber membranes tailored to different application requirements.

## 2. Gelatin

### 2.1. Source of Gelatin

**Figure 2:**
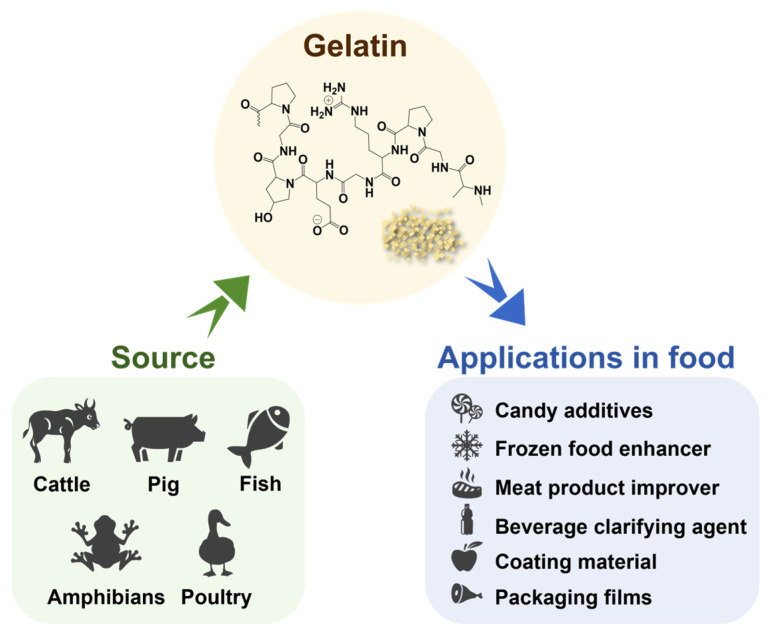
Sources of gelatin and its applications in food industry.

Gelatin, a high-molecular-weight hydrocolloid composed of 18 amino acids, is colorless, tasteless, and widely used in various applications. It is derived from the hydrolytic degradation of collagen extracted from tissues such as mammalian skin, bones, and tendons. Presently, cattle and pigs are the primary sources of gelatin [15]. In addition, with the advancement in resource exploration and utilization, the sources of gelatin have diversified (Figure 2) to include varieties from fish, amphibians, and poultry [16,17,18].

The content of imino acid varies among gelatin from different fish species, with warm-water fish containing higher levels than cold-water fish [19]. Therefore, gelatin extracted from warm-water fish—characterized by elevated proline and hydroxyproline levels—has been shown to enhance the mechanical properties of thin films, including tensile strength, elongation at break, and water vapor permeability [20]. Additionally, Sarbon et al. found that chicken skin gelatin has better gel strength and stability than cow skin [21].

The extraction of natural gelatin typically employs three main processes: acidic, alkaline, and enzymatic [15,22]. In various processing methods, the chain structure of gelatin molecules depends on factors such as gelatin concentration, temperature, and the energy required for the formation of secondary structures, determining differences in spatial arrangements and intermolecular interactions between chains [23]. Based on different pre-treatment processes, gelatin can be categorized into type A and type B. Type A gelatin, with an isoelectric point of 6–9, is usually prepared from acid-treated porcine skin collagen, while type B gelatin, with an isoelectric point of approximately 5, is derived from alkaline-treated bovine skin collagen [15,24]. Notably, the alkaline pre-treatment (type B) of collagen protein to form gelatin involves the hydrolysis of the amide groups of asparagine and glutamine into carboxyl groups while acidic pre-treatment (type A) barely affects the amide groups [25]. However, acid–alkali processes have drawbacks, including producing significant waste, using raw materials inefficiently, breaking non-covalent bonds of gelatin, and its triple helical structure [26]. In addition to acid–alkali process extraction, gelatin can also be extracted using protein hydrolysis enzymes [22]. Compared to acid-based methods, enzymatic extraction offers higher yield, shorter processing time, and generates less waste. However, the high temperatures involved in enzymatic extraction can degrade gelatin’s α and β chains, compromising its quality and gel strength. Nevertheless, it was reported that enzymatic gelatin extraction can enhance yield without compromising quality under the influence of ultrasound, offering a potential solution to balancing quality and yield issues associated with enzymatic collagen extraction [27].

### 2.2. Properties and Applications of Gelatin

The essential structure of gelatin can be described as a mixture of α-chains (one polymer/single chain), β chains (two α-chains covalently crosslinked), and γ chains (two α-chains covalently crosslinked) [24,28]. One of the important physical properties of gelatin is gel strength, which depends largely on the pre-treatment and extraction process, also known as bloom value [15]. Typically, a bloom value less than 150 is considered low bloom, 150–220 is moderate bloom, and 220–300 is high bloom. A higher bloom value indicates greater strength and stiffness of gelatin [24]. Meanwhile, the viscosity of gelatin is also an important indicator, which is influenced by factors such as gelatin concentration, molecular weight, molecular size distribution, and gelatin pH [18]. Due to gelatin exhibiting thermal reversibility, gelatin should be stored at low temperatures, where it can maintain its shape and conformation for extended periods [15]. Additionally, gelatin demonstrates high biocompatibility, low antigenicity, low viscosity, and water retention properties [29]. Due to the versatility of gelatin, it has found wide applications in biomedical [25,30], photographic [28], cosmetic [31], and food industries [6,32]. Additionally, gelatin plays a role in the conservation and restoration of cultural heritage artifacts [33,34].

The widespread application of gelatin in the food industry (Figure 2) is primarily attributed to its excellent gelation, thickening, and stabilizing properties, which contribute to enhancing the taste, texture, and appearance of food products. For instance, in beef burger formulations, combining gelatin with soluble dietary fiber derived from seed powder as a novel fat replacer not only enhances the nutritional of the product but also elevates its taste and chewiness [35]. In candies and jelly products, leveraging the gelation properties of gelatin can impart a certain degree of viscosity and chewiness [36]. In addition, gelatin is also valued for its outstanding mechanical and barrier properties, making it a preferred material for researchers in the development of food packaging materials and films [17,37,38]. For instance, in certain cooked meat products, gelatin has been utilized as a film or coating material to enhance texture and extend the shelf life of the food [7,39]. Moreover, gelatin exhibits effective water retention properties, serving as a protective barrier against moisture loss in certain vegetables and fruits [5]. Gelatin can also be combined with natural compounds to prepare active food packaging, enhancing microbial protection capabilities and improving the mechanical properties of food packaging [40]. Therefore, conducting in depth research and applying gelatin in food packaging holds significant importance for improving food quality and advancing human health.

## 3. Electrospinning

### 3.1. Principle of Electrospinning and Applications for Food Packaging

**Figure 3:**
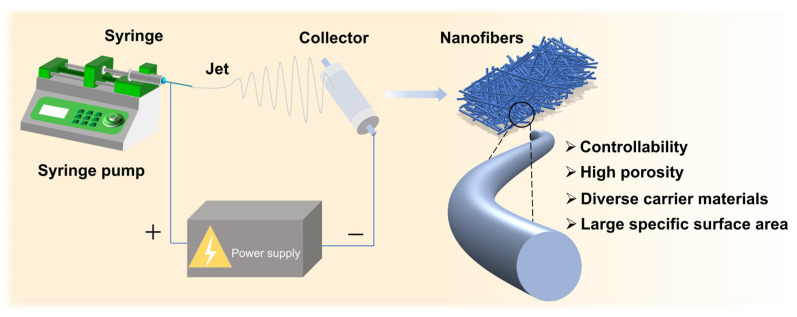
Schematic illustration of electrospinning process.

Electrospinning is an efficient and cost-effective nanofiber fabrication technique (Figure 3). Electrospinning devices typically consist of a high-voltage power supply, a liquid delivery system (including syringe pumps, syringes, and nozzles), and a collector. During the electrospinning process, a polymer solution or melt is first loaded into the syringe, and the liquid is pushed to the nozzle to form spherical droplets. Subsequently, a specific electric field is applied at the nozzle by applying high voltage and low current, while the collector is grounded or with lower electric field. Then, the polymer solution is propelled at a constant speed using a syringe pump. When the electrostatic forces acting on the polymer solution exceed its surface tension, a Taylor cone forms at the nozzle tip, and a charged jet is ejected. This process is accompanied by solvent evaporation as the jet travels through the air, ultimately leading to the solidification and deposition of nanofibers onto the collector. The resulting nanofibers typically exhibit diameters ranging from tens to hundreds of nanometers [37,41,42].

Electrospinning technology has garnered significant attention from researchers due to its high efficiency, good controllability, diverse carrier materials, and the resulting nanofibers’ high porosity and large specific surface area. These properties enable broad applications across diverse fields, including biomedicine (e.g., tissue engineering scaffolds [43], drug delivery systems [44]), environmental engineering (e.g., air/water filtration membranes [45]), energy storage (e.g., battery separators [46]), and flexible electronics (e.g., wearable sensors [47,48]). To obtain high-quality nanofibers through electrospinning, it is essential to carefully regulate several key parameters, such as polymer solution concentration, polymer molecular weight, solution viscosity, voltage, distance between the nozzle and collector, and environmental conditions. These influencing factors collectively determine the overall morphology of the fibers. For instance, Vince Beachley [49] elucidated the factors affecting the diameter of nanofibers such as polymer concentration, process parameters, and voltage magnitude. Generally, fiber diameter typically exhibits an inverse correlation with applied voltage, primarily attributed to the enhanced jet stretching driven by elevated electric field intensity. However, exceeding critical voltage thresholds induces progressive jet instability, characterized by stochastic bead formation and diameter heterogeneity. Concurrently, suboptimal needle-to-collector distances promote incomplete jet solidification, resulting in discontinuous bead-on-string morphologies rather than uniform fibrous architectures [50,51]. Overall, by optimizing and controlling the electrospinning process parameters, well-shaped and high-performance nanofiber membranes can be prepared.

In recent years, electrospun nanofiber membranes have demonstrated a promising development trend and enormous potential in food packaging and preservation, as presented in the VOSviewer-based visualization analysis result (Figure 1) [12,13]. Traditional food packaging technologies, such as film extrusion, typically exhibit high water vapor transmission rates, while solution casting is characterized by poor mechanical strength. Spray coating, on the other hand, often offers limited functionality. Compared to these conventional methods, electrospinning technology provides notable advantages, including superior water vapor barrier properties, enhanced mechanical strength, and greater functional versatility. These advantages arise from the unique structural characteristics of electrospinning nanofibers, including their high surface area, increased porosity, and ability to effectively incorporate functional agents. As a result, electrospun membranes demonstrate superior performance in terms of barrier properties, mechanical flexibility, and active functionalities. For instance, antimicrobial agents and antioxidants can be incorporated into electrospun nanofibers to impart or enhance the antimicrobial and antioxidant properties of food packaging [52,53,54]. By carefully selecting and optimizing the electrospinning composition and process, food packaging can also acquire edibility [37], hydrophobicity [55], thermal stability, deformation resistance, etc. [56,57]. Additionally, intelligent food packaging prepared using electrospinning can respond to and monitor conditions such as pH, temperature, oxygen, etc., providing an additional layer of protection for food safety.

In food packaging applications, coaxial electrospinning is prioritized for scenarios requiring the sustained release of active ingredients or the protection of sensitive substances, as its core–shell structure enables effective encapsulation and controlled delivery. Emulsion electrospinning is suitable for co-loading hydrophobic and hydrophilic components to achieve synergistic functionalities, though careful consideration of component compatibility and emulsion stability is essential. Uniaxial electrospinning is typically reserved for moisture barriers, physical protection, or short-term antimicrobial applications, or as a foundational substrate combined with complementary technologies. These three electrospinning methods are briefly described below.

### 3.2. Common Types of Electrospinning in Food Packaging

**Figure 4:**
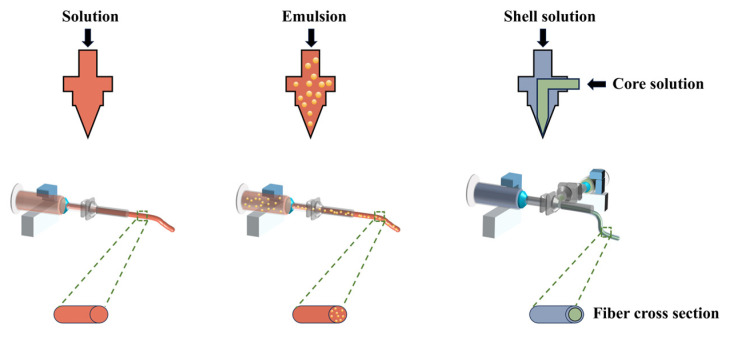
Common types of electrospinning for food packaging.

Up to now, electrospinning technology has evolved various spinning methods, primarily including single-nozzle, coaxial, and emulsion electrospinning for food packaging applications (Figure 4). It is essential to consider different factors such as polymer solution properties, types of active factors, application scenarios, and spinning environments to employ various spinning techniques for achieving diverse structures and functionalities for food packaging applications (Table 1).

#### 3.2.1. Single-Nozzle Electrospinning

The single-nozzle electrospinning technique represents a straightforward approach for the fabrication of nanofibers. This method entails the application of high-voltage electricity to a polymer solution within a conventional single-nozzle, inducing the formation of a charged jet that elongates into nanofibers under the influence of an electric field [67]. Within the realm of food packaging, some natural polymers or proteins such as starch, zein, and chitosan are frequently employed for the production of biodegradable packaging materials [58,68,69,70]. Moreover, the single-nozzle electrospinning technique enables the incorporation of active factors into polymer solutions, thereby facilitating the fabrication of active food packaging nanofiber membranes. For instance, researchers [58] developed a biodegradable and antimicrobial food packaging material by incorporating an antimicrobial cocktail into zein solution using a direct solution integration method. Similarly, Liu et al. synthesized antimicrobial food packaging materials through electrospinning utilizing a polylactic acid (PLA)/tea polyphenol solution [59].

However, nanofibers synthesized via single-nozzle electrospinning typically exhibit relatively simple structures, posing challenges in the construction of multifunctional fiber membranes. When incorporating multiple active components into the polymer solution for electrospinning, it is crucial to consider their interactions within the solution as well as their release kinetics under varying environmental conditions. One promising approach to address this issue involves the fabrication of nanofibers bearing active functional groups, followed by the loading of active factors into the nanofiber membrane through chemical modification to achieve controlled release. For instance, Liu et al. [71] synthesized nanofiber membranes with photothermal synergistic antibacterial properties, antioxidative capabilities, and excellent biocompatibility through chemical modification methods. Furthermore, subsequent physical or chemical crosslinking of single nanofibers can also achieve certain modifications. For example, Yang et al. [60] prepared a porous structure of food packaging nanofibers by combining safflower seed meal with eliminable pullulan, followed by crosslinking with cinnamaldehyde. By embedding active components within these porous nanofibers or regulating their controlled release, this method presents a promising pathway for the development of next-generation edible food packaging.

#### 3.2.2. Coaxial Electrospinning

In order to enhance the quality and functionality of electrospinning nanofibers, two nozzles can be coaxially combined to enable the spinning of two different solutions, thus constructing multifunctional nanofibers. Coaxial electrospinning [72] involves loading two polymer solutions with specifically tailored viscosity matching into separate independent syringes, which are then advanced at precisely regulated flow rate ratios. As the solutions converge at the coaxial nozzle, controlled miscibility characteristics between the two phases enable interfacial stabilization. Under a high-voltage electric field, this stabilized system forms a compound Taylor cone, thereby producing fibers with well defined core–shell architectures. Coaxial electrospinning technique often serves to complement the properties and functionalities of fibers, such as enhancements in mechanical performance [73,74,75], stability [76], electrical conductivity [77], the conferment of thermal regulation capabilities into the fibers [78], the improved control of drug release [79], and the adsorption capacity of nanofibers [80].

Presently, coaxial electrospinning has found extensive application across various domains [81] including biomedicine (e.g., controlled release of active factors, tissue engineering [82,83,84]), electrochemistry/electrical engineering (e.g., battery separators [85], photovoltaic materials [86]), and environmental protection (air filtration [87], wastewater treatment [76]). Although specific treatments can be applied to nanofibers produced by single-nozzle electrospinning to achieve the delayed release of bioactive factors, the risk of premature release remains a challenge. Nevertheless, preparation of shell–core nanofibers by coaxial electrospinning is a promising solution to this issue. For instance, Liu et al. [79] fabricated a Janus nanofiber wound dressing with one coaxial nanofiber layer loaded with the phase change material lauric acid in the shell layer and anti-inflammatory ibuprofen in the core layer, thereby achieving the controlled release of ibuprofen by the temperature-sensitive phase change characteristic of lauric acid. Beyond wound dressing applications, coaxial electrospinning has also been explored for encapsulating active ingredients in food packaging, thereby extending food freshness and shelf life. Han et al. [61] prepared a coaxial antibacterial nanofiber with a core layer containing a mixed solution of cinnamaldehyde and tea polyphenols, and a shell layer made of PLA, exhibiting strong hydrophobicity, excellent thermal stability, and mechanical properties, which can effectively destroy the cell membrane of Shewanella putrefaciens, thereby extending food shelf life.

Notably, in coaxial electrospinning, the intricate design and process factors (such as the flow rate of the core–shell fluids, differences in conductivity and viscosity among different polymer solutions) may potentially impact the target fibers, limiting the application of coaxial electrospinning in food packaging [13,81].

#### 3.2.3. Emulsion Electrospinning

In recent years, the emulsion electrospinning technique has rapidly evolved and is widely regarded as a novel method capable of one-step preparation of nanostructures with core–shell architecture. The application prospects of emulsion electrospinning technology are extensive [88], encompassing various domains such as medical [89,90,91], environmental protection [87], textiles [92], and energy storage [93]. This technique is primarily employed in two-phase dispersion systems, namely oil-in-water (O/W) or water-in-oil (W/O), where hydrophilic or hydrophobic active factors are directly encapsulated into the core–shell fibers, resulting in the formation of nanostructures with core–shell architecture. Under the influence of a high-voltage electric field, the evaporation rate of the outer emulsion exceeds that of the inner emulsion, causing the emulsion to condense into elliptical shapes deposited on the surface of objects [94,95]. Due to the ability of emulsion electrospinning technology to dissolve or disperse active factors in emulsions, the resulting nanostructured core–shell fibers effectively prevent the burst release of active factors, thus improving the stability and release performance of the active factors. For instance, Dede et al. [65] successfully encapsulated basil oil in a water-in-oil emulsion of zein/alginate to prepare core–shell nanofiber food packaging by emulsion electrospinning, which exhibited the diffusion release characteristics of basil oil. Beyond direct encapsulation of active factors in emulsions, further optimization of release performance can be achieved by modifying the crystalline structure of electrospun nanofibers. Bruni et al. [66] encapsulated β-carotene in a mixed emulsion of soy protein isolate and polyvinyl alcohol for electrospinning, due to the partial collapse of the fiber structure during annealing, resulting in reduced porosity of the matrices and formation of polyvinyl alcohol crystallites, helping achieve a slower and longer lasting release of β-carotene.

It is worth noting that emulsion stability plays a critical role in the preparation of nanofibers. The stability of emulsions is influenced by various factors, including flocculation, coalescence, and gravitational separation. However, the introduction of emulsifiers, thickeners, and gelling agents can induce the formation of droplet clusters and gels, thereby enhancing the stability of emulsions [94,96]. Additionally, studies have shown that different types and concentrations of emulsifiers may have varying effects on the morphology, mechanical properties, and functional groups of electrospinning nanofibers [13,97], underscoring the significance of selecting appropriate emulsifiers for the synthesis of target nanostructured fibers.

## 4. Functional Strategies for Gelatin-Based Electrospun Food Packaging

Due to the increasing emphasis on food safety, the demand for food packaging has evolved. Apart from serving as storage containers, there is a growing desire for packaging to possess properties such as antioxidation, antimicrobial activity, and edibility. Known for their excellent mechanical and barrier properties, gelatin-based films hold significant potential in biodegradable food packaging [17]. Compared with traditional biomaterials such as chitosan, cellulose, and starch, electrospun gelatin-based nanofibers demonstrate distinct advantages. Their unique amino acid composition and molecular flexibility confer exceptional structural tunability, enabling precise customization of barrier properties through controlled morphological characteristics including fiber diameter and porosity. Functionally, gelatin’s active amino groups facilitate direct covalent conjugation with antioxidants and bioactive compounds without chemical modification [38], whereas starch or cellulose requires additional functionalization for comparable efficacy. Furthermore, gelatin’s multi-solvent compatibility supports diverse electrospinning techniques (e.g., emulsion and coaxial electrospinning), achieving stable encapsulation of volatile active compounds—a processing versatility superior to rigid biomaterials like chitin. The integration with chitosan generates synergistic antibacterial effects, exhibiting functional superiority over single-component systems through enhanced antibacterial performance [98]. The barrier performance of gelatin electrospun nanofibers is governed by their structural morphology (e.g., fiber, diameter, porosity) and chemical composition. The highly tortuous three-dimensional interwoven architecture of nanofibrous networks imposes substantial resistance to water vapor diffusion. Additionally, submicron-scale pores (<1 μm) act as physical barriers against microbial penetration, while bioactive agents (e.g., essential oils) embedded within fibers enable sustained antimicrobial activity [37]. The barrier performance of the films also can be significantly enhanced through the hydrophilic–hydrophobic modulation strategy of the gelatin matrix, which includes the introduction of polymer blending components (e.g., PLA [99]) or the addition of lipid-based modifiers (e.g., beeswax [100]). In comparison to traditional extrusion casting processes, electrospinning presents greater advantages in the preparation of gelatin-based films. Electrospun gelatin-based nanofibrous membranes not only inherit the good properties of gelatin-based films fabricated by conventional processes, but also are easy for loading active substances, thereby meeting the multifunctional requirements of food packaging [12]. In recent years, numerous studies have focused on the functional gelatin-based electrospun nanofiber membranes obtained through modification and their applications for food packaging. The following is a categorical review and discussion of this research.

### 4.1. Hydrophobic Modification

**Figure 5:**
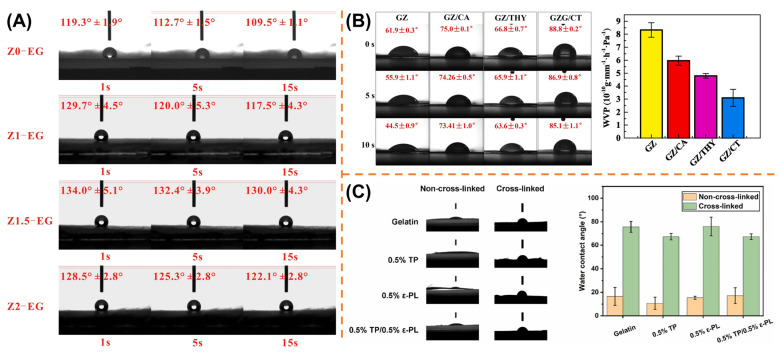
Methods of hydrophobic modification of gelatin-based electrospun nanofibers for food packaging applications. (**A**,**B**) Incorporation of active substances such as zinc oxide nanoparticles (**A**), cinnamaldehyde, and thymol (**B**). Reprinted with permission from references [103,104]. Copyright 2018 American Chemical Society and 2023 Elsevier. (**C**) Integration of electrospinning with crosslinking. Reprinted with permission from reference [105]. Copyright 2022 Elsevier.

Moisture is one of the pivotal factors affecting food deterioration and spoilage. Elevated water activity not only promotes microbial proliferation but also accelerates lipid oxidation and enzymatic degradation. Hydrophobic performance is a good indicator to measure food packaging materials [101]. Hydrophobic packaging plays a dual protective role [102]: (1) acting as a barrier against external humidity penetration to suppress microbial growth, and (2) preventing structural dissolution of the packaging matrix itself. Consequently, controlling moisture migration through advanced packaging systems is critical for shelf life extension. Although gelatin-based materials are inherently hydrophilic, emerging strategies such as electrospinning with hydrophobic additives or multilayer composite designs have demonstrated success in reducing water vapor permeability while maintaining mechanical integrity (Figure 5). These innovations shift gelatin’s hydrophilicity towards hydrophobicity without compromising its biodegradability, offering sustainable solutions for moisture-sensitive food preservation.

#### 4.1.1. Incorporation of Hydrophobic Compounds

Incorporating appropriate hydrophobic substances is a viable method for enhancing the water barrier capability of gelatin-based electrospun nanofiber membranes. It was reported that the water contact angle of gelatin/chitosan/3-phenyllactic acid electrospun nanofibers reached its maximum when 3-phenyllactic acid was incorporated at a concentration of 2% [99]. This was because, at this concentration, the carboxyl groups of 3-phenyllactic acid reacted with the hydroxyl groups of chitosan to form insoluble hydrogen bonds. PLA films exhibit hydrophobicity [106]. However, PLA films coated with gelatin–chitosan electrospun nanofiber membranes containing nisin exhibited lower water vapor permeability [107]. This was due to the barrier effect of the nanofiber coating and the dense layer formed by the interaction of the negatively charged regions of nisin and gelatin. Gelatin-based electrospun nanofiber membranes doped with chitosan, PLA, and betel leaf ethanolic extract has also been demonstrated with excellent water barrier properties [108]. In addition to the inherent hydrophobic nature of PLA and the physical barrier effect of the dense nanofiber membrane formed by gelatin–chitosan crosslinking, the incorporation of betel leaf ethanolic extract also enhanced the water vapor barrier capability of the nanofiber membrane. Furthermore, incorporating ε-polylysine into gelatin/chitosan nanofiber membranes has also been reported to further enhance the water resistance of the membranes [109].

Zein, often co-spun with gelatin, contributes to nanofiber membranes displaying notable hydrophobic properties [110,111]. Incorporating zein nanofibers within the casted gelatin film showed that the hydrophobicity of gelatin composite films increased with the rise in zein concentration [112]. On the basis of zein, other active substances can be incorporated to further enhance the hydrophobicity of electrospun gelatin-based nanofiber membranes. As the incorporation ratio of perillaldehyde increased, the proportion of hydrophobic groups enhanced, leading to a transition of electrospun gelatin–zein–perillaldehyde nanofiber membranes from hydrophilic to hydrophobic [113]. Other substances, including thymol, ε-PL, gallic acid, eugenol, resveratrol, catechin, and zinc oxide nanoparticles (ZnO NPs), were also found to enhance the water barrier properties of gelatin–zein electrospun nanofiber membranes [38,114,115,116,117].

The incorporation of essential oil (EO) can significantly alter the hydrophilicity of electrospun gelatin-based nanofiber membranes, rendering them hydrophobic. The successful loading of angelica EO enhanced the hydrophobic and moisture-resistant properties of electrospun gelatin-based nanofibrous membranes, thereby improving their efficacy as food packaging [118]. Chamomile EO, peppermint EO [37], thymol, which is a major component of thyme EO [104], *Cuminum cyminum* EO [119], and *Oliveria decumbens Vent* EO [120] have also been reported to improve the water barrier performance of gelatin-based electrospun nanofiber membrane. Furthermore, the incorporation of EOs with nanoparticles also exhibits excellent modification effects. Shanbehzadeh et al. found that the successful loading of cumin EO and ZnO NPs can enhance the overall hydrophobicity of electrospun polycaprolactone-gelatin nanofiber membrane [121]. Similarly, the incorporation of *Zataria multiflora* EO (ZEO) and ZnO NPs reduced the overall hydrophilic group ratio, thereby enhancing hydrophobicity of the electrospun gelatin-based nanofiber membrane surface [122].

In addition to the aforementioned substances, there are other substances capable of improving the hydrophobicity of electrospun gelatin-based nanofiber membranes. Ethylcellulose has been incorporated into electrospun gelatin-based nanofiber membranes, and the membrane exhibited hydrophilicity when gelatin predominated and hydrophobicity when ethylcellulose predominated [123]. Furthermore, the addition of ZnO NPs was also reported to enhance the surface hydrophobicity of electrospun ethylcellulose-gelatin nanofiber membrane [103]. Moreover, cellulose acetate [124], pine-honey [125], and probiotics [126] have also been incorporated into electrospun gelatin-based nanofiber membranes to enhance the water resistance of the nanofiber membranes.

#### 4.1.2. Hybrid Processing with Auxiliary Techniques

In addition to electrospinning doped with active substances, the enhancement of the hydrophobicity of electrospun gelatin-based nanofiber membranes can also be achieved by integrating electrospinning with other techniques to produce composite films, multilayer films, or crosslinked membranes.

Wang et al. prepared a three-layer film consisting of gelatin-propyl gallate-gelatin, where the outer and inner layers of gelatin were fabricated using electrospinning and solvent casting methods, respectively [127]. And the results revealed that, due to the lower water permeability of solvent casted gelatin films, the entire composite film still retained a certain level of water vapor barrier capability.

Crosslinking has been previously demonstrated to enhance the mechanical properties and hydrophobicity of electrospun gelatin-based nanofibers [128]. As early as 2014, Simon et al. found that glucose could increase the degree of gelatin crosslinking, and successfully prepared thermally crosslinked gelatin scaffolds using glucose as the crosslinking agent [129]. The Maillard reaction essentially involves the reaction between carbonyl compounds (reducing sugars) and amino compounds (amino acids and proteins). In the thermal crosslinking process of gelatin with glucose, the Maillard reaction occurred, wherein glucose functioned as the reducing sugar, while the amino acid residues in gelatin proteins acted as the amino compounds. Deng Lingli et al. prepared thermally crosslinked electrospun gelatin–zein nanofibers with glucose through the Maillard reaction, resulting in nanofiber membranes with enhanced surface hydrophobicity [130]. Subsequent studies also demonstrated that electrospun gelatin-based nanofiber membranes crosslinked with glucose through the Maillard reaction exhibited higher surface hydrophobicity [131,132]. Moreover, in addition to glucose, thermal crosslinking treatment using citric acid or fructose as crosslinking agents can also enhance the water barrier properties of electrospun gelatin-based nanofiber membranes [98,133]. Furthermore, Lan et al. found that enhancing the water resistance of electrospun gelatin-based nanofiber membranes could also be achieved without using crosslinking agents but through dehydrothermal treatment [105]. Apart from thermal crosslinking, Schiff base crosslinking has also been shown to be a viable method to improve the water barrier properties of electrospun gelatin-based nanofiber membranes [134].

In summary, the hydrophobic modification of gelatin-based electrospun nanofibers has been systematically achieved through two complementary strategies: (1) the incorporation of functional additives and (2) hybrid processing with auxiliary techniques. By integrating hydrophobic compounds such as zein, essential oils, ε-polylysine, and nanoparticles (e.g., ZnO), the water barrier properties of nanofiber membranes are enhanced through mechanisms including hydrogen bonding, hydrophobic group enrichment, and dense structural formation. Concurrently, hybrid approaches—such as multilayer film fabrication, thermal crosslinking via the Maillard reaction, and dehydrothermal treatments—further optimize moisture resistance without compromising biodegradability. These modifications effectively address gelatin’s inherent hydrophilicity, enabling tailored control over water vapor permeability for moisture-sensitive food preservation. The versatility of additive selection (e.g., plant extracts, polymers, crosslinkers) and processing innovations (e.g., solvent casting-coupled electrospinning) demonstrates the adaptability of gelatin-based systems to meet diverse packaging requirements. In addition, the water contact angle is often used as an index of hydrophobicity in current studies. In the future, multiple performance parameters such as water contact angle and water vapor transmission should be paid attention to at the same time, which can improve the scientific rigor of research and prevent errors caused by complex factors. For example, essential oils may plasticize the film or change the surface roughness, which can affect the water contact angle to some extent.

### 4.2. Functionalization for Edible Packaging

In light of the negative environmental consequences of the extensive use of traditional food packaging, edible food packaging emerges as a novel and rational solution [135]. Typically prepared from food-grade polymers such as proteins, lipids, and polysaccharides, edible food packaging offers key advantages, including renewability, biodegradability, and biocompatibility [136]. In recent years, gelatin has emerged as a promising material for the production of edible film packaging [40]. However, pure gelatin exhibits limited bioactivity. To enhance its properties, it is common practice to incorporate other natural products with similar characteristics, such as biodegradability, biocompatibility, and non-toxicity, through co-electrospinning to produce edible food packaging with superior performance. For instance, chitooligosaccharide is a naturally non-toxic, easily absorbable oligosaccharide obtained from the degradation of chitosan. Gelatin-based electrospun nanofiber membranes loaded with chitooligosaccharide demonstrated good hydrolytic degradation stability, outstanding antioxidant and antibacterial properties, and a remarkable ability to extend the freshness of crucian carp when used as coatings, showcasing their immense potential in edible food packaging [132].

Considering the safety of food packaging, natural plant extracts are also frequently employed as additives in gelatin-based electrospun nanofibers. Among these, EOs derived from plants are widely used. Electrospun gelatin-based nanofiber membranes loaded with peppermint EO and chamomile EO exhibited excellent biological activity and cell-friendly characteristics, making them a prospective candidate material for the development of edible food packaging [37]. Wrapping angelica root (Angelica sylvestris) oil in zein/hyaluronic acid/gelatin-based fibers through emulsion electrospinning allowed for the preparation of edible food packaging with good mechanical properties and antimicrobial capabilities [137]. Beyond food packaging, electrospun Plantago psyllium seed gum-gelatin-Cuminum cyminum EO nano-composite materials was prepared to be applied as edible coatings for food [119]. Plant-derived protein extracts also showed significant potential for edible food packaging. In a previous study, a 5% *w*/*w* electrospun gelatin/glycerol monolaurate microemulsion super-hydrophilic nanofiber membrane was developed, showing potential in soluble and edible food packaging [138]. Similarly, a needleless electrospun Spirulina protein concentrate-gelatin nanofiber membrane was shown to dissolve rapidly in acidic, alkaline, and neutral aqueous solutions, making it suitable for edible packaging applications in nutraceutical delivery [139]. Moreover, various bioactive compounds naturally present in plants, such as food protein zein, cinnamaldehyde, thymol, perillaldehyde, and ε-PL, have also been successfully incorporated into electrospun gelatin-based edible food packaging [104,114].

In general, the integration of gelatin with natural additives through electrospinning represents a strategic approach to develop edible packaging materials. This synergistic design reduces the dependence on pure gelatin formulations while maintaining or enhancing material performance through complementary interactions. By lowering the gelatin content required per unit of packaging, such composite systems could proportionally decrease the industrial demand for collagen-derived raw materials sourced from livestock. This indirect mitigation of agricultural resource consumption aligns with sustainable development goals in food packaging technology. Future efforts should prioritize exploring diverse additive–gelatin combinations to establish adaptable frameworks that balance functional requirements, edibility standards, and environmental sustainability in food preservation applications.

### 4.3. Antibacterial Functionalization

**Figure 6:**
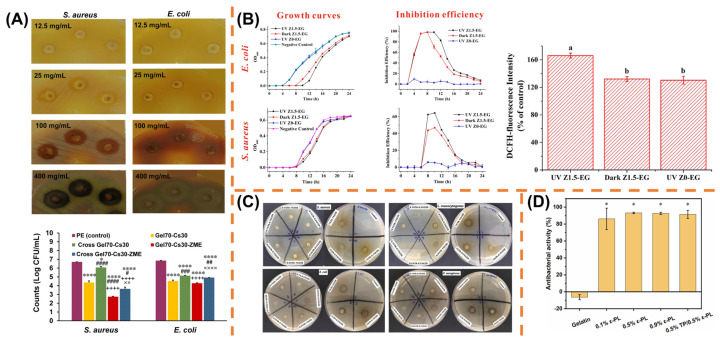
Some representative studies of electrospun gelatin-based nanofibers incorporated with antibacterial agents. (**A**) Inhibition against *S. aureus* and *E. coli* by different concentrations of electrospun chitosan–gelatin nanofibers containing *Zataria multiflora* extract. Reprinted with permission from reference [98]. (****: *p* < 0.0001 and *: *p* < 0.05 vs. PE, ####: *p* < 0.0001, ###: *p* < 0.001, ##: *p* < 0.01 and #: *p* < 0.05 vs. Gel70-Cs30, ++++: *p* < 0.0001 vs Cross Gel70-Cs30, ××: *p* < 0.01 and ××××: *p* < 0.0001 vs. Gel70-Cs30-ZME.) Copyright 2024 Springer. (**B**) Inhibition against *E. coli* and *S. aureus* of electrospun ethylcellulose-gelatin nanofibers containing zinc oxide nanoparticles. Lowercases indicate statistical significance (*p* < 0.05). Reprinted with permission from reference [103]. Copyright 2018 American Chemical Society. (**C**) Inhibition zones of PLA films coated with gelatin–chitosan nanofibers incorporated with tannic acid and chitooligosaccharides. Reprinted with permission from reference [156]. Copyright 2022 Elsevier. (**D**) Antibacterial activity of electrospun gelatin/tea polyphenol/ε-PL nanofiber membranes. Reprinted with permission from reference [105]. Copyright 2022 Elsevier.

Microbial contamination is one of the primary causes of food spoilage. To address this issue, the development of antimicrobial food packaging has emerged as a promising solution [140]. In recent years, electrospinning has shown significant advancements in the fabrication of antimicrobial materials [141]. It allows for the incorporation of antimicrobial agents into fibers, imparting excellent antibacterial properties to the electrospun membranes [52]. Gelatin, as an environmentally friendly polymer, is frequently utilized as a substrate material for the preparation of active food packaging [142]. Consequently, there has been growing attention towards the development of gelatin-based electrospun antimicrobial food packaging. The following are summarized and discussed according to the four categories of antibacterial agents incorporated into electrospun gelatin-based nanofibers (Figure 6). Moreover, antimicrobial substances applied in electrospun gelatin-based nanofibrous antibacterial food packaging are summarized in Table 2.

**Table 2:** Antimicrobial substances applied in electrospun gelatin-based nanofibrous antibacterial food packaging.

Categories	Active Substance	Targeted Microbes	References
EO	*Mentha spicata* L. EO	*S. aureus*, *L. monocytogenes*, *Bacillus subtilis*, *Bacillus cereus*, *Salmonella enterica*, *E. coli*	[143,144]
	Peppermint EO	*E. coli*, *S. aureus*	[37]
	Chamomile EO	*E. coli*, *S. aureus*	[37]
	*Zataria multiflora* EO (*Zataria multiflora* extract)	*S. aureus*, *Bacillus cereus*, *L. monocytogenes*, *E. coli*, *Salmonella typhimurium*, *Aspergillus niger*, *Penicillium notatum*	[98,122,145]
	Cinnamon zeylanicum EO (Cumin EO)	*S. aureus*, *Bacillus cereus*, *L. monocytogenes*, *E. coli*, *Salmonella typhimurium*	[119,121,145]
	Angelica EO (Angelica root oil)	*E. coli*, *S. aureus*, *Salmonella enteritidis*, *L. monocytogenes*, *Lactobacillus rhamnosus*	[118,137]
	*Oliveria decumbens Vent* EO	*E. coli*, *S. aureus*	[120]
	Ginger EO	*E. coli*, *S. aureus*	[146]
	Perillaldehyde	*S. aureus*, *Salmonella enterica*	[113,114]
	Cinnamaldehyde	*E. coli*, *S. aureus*, *Aspergillus niger*, *L. monocytogenes*	[104,147]
EO	Thymol	*S. aureus*, *L. monocytogenes*, *Salmonella enterica*	[104,114]
	Thyme EO	*Campylobacter jejuni*	[148]
	Carvacrol	*Pseudomonas aeruginosa*, *Shewanella putrefaciens*, *E. coli*, *S. aureus*, *Salmonella enterica* serovar Typhimurium 14028s	[149,150]
	Eugenol	*E. coli*, *S. aureus*, *Aspergillus niger*, microorganism of beef	[116,124,147,151,152]
	Limonene	*E. coli*, *S. aureus*, *Aspergillus niger*	[147]
Nanoparticles	Zinc oxide nanoparticles	*E. coli*, *S. aureus*, *Aspergillus niger*, *Penicillium notatum*, *Botrytis cinerea*	[103,117,121,122]
	Eugenol nanoparticles	*E. coli*, *S. aureus*	[151]
	Moringa oil-loaded chitosan nanoparticles	*Lactobacillus plantarum*, *S. aureus*	[153]
Nanoparticles	Thyme EO/β-cyclodextrin /ε-polylysine nanoparticles	*Campylobacter jejuni*	[148]
Chitosan and its derivatives	Chitosan	*E. coli*, *S. aureus*, *Klebsiella pneumoniae*, *Salmonella Enteritidis*, *Pseudomonas aeruginosa*, *L. monocytogenes*	[98,99,109,154,155]
	Chitooligosaccharide	*E. coli*, *Vibrio parahaemolyticus*, *Pseudomonas aeruginosa*, *S. aureus*, *L. monocytogenes*	[132,156]
Other antibacterial agents	Nisin	*S. aureus*, *L. monocytogenes*, *Pseudomonas aeruginosa*, *E. coli*	[107]
	Fucoxanthin	*E. coli*, *S. aureus*	[157]
	Sage extract	*E. coli*, *S. aureus*	[158]
	Propolis	*E. coli*, *S. aureus*	[134]
	Resveratrol	*E. coli*, *S. aureus*	[38]
	Lauroyl arginine ethyl	*E. coli*, *S. aureus*	[159]
	Allyl isothiocyanate	*E. coli*, *S. aureus*	[160]
Other antibacterial agents	Glycerol monolaurate	*E. coli*, *S. aureus*	[138]
	Butylated hydroxyanisole	*S. aureus*, *Rhizopus* sp., *Mucor* sp., *Aspergillus* sp., *Penicillium* sp.	[161]
	Probiotics	*Vibrio parahaemolyticus*, *Salmonella Typhimurium*, *S. aureus*, *L. monocytogenes*	[126]
	Curcumin	*E. coli*, *S. aureus*	[155]
	Tannic acid	*E. coli*, *Pseudomonas aeruginosa*, *S. aureus*, *L. monocytogenes*	[156]
	ε-polylysine	*E. coli*, *S. aureus*, *Klebsiella pneumoniae*, *Salmonella Enteritidis*, *Pseudomonas aeruginosa*, *L. monocytogenes*, *Campylobacter jejuni*, *Shewanella putrefaciens*	[105,109,114,115,148,162]

#### 4.3.1. EOs Encapsulation

Due to the presence of phenolic groups, EOs exhibit excellent antimicrobial, antifungal, and antioxidant properties [163]. EOs are often encapsulated as antimicrobial agents in electrospun nanofibers, inhibiting the growth of bacteria or fungi and prolonging the shelf life of food [53].

Due to the sustained antimicrobial activity of *Mentha longifolia* L. EO, gelatin-based electrospun nanofibers encapsulating *Mentha longifolia* L. EO exhibited significant inhibitory effects against *Staphylococcus aureus* (*S. aureus*) and *Listeria monocytogenes* (*L. monocytogenes*), effectively extending the shelf life of fresh trout filets [143]. Apart from these two bacteria, *Mentha longifolia* L. EO also demonstrated its effective inhibition against *Escherichia coli* (*E. coli*), *Salmonella enterica*, *Bacillus subtilis*, *and Bacillus cereus* in other reported electrospun gelatin membranes [144].

*ZEO* exhibits remarkable antibacterial properties. Soy protein isolate–gelatin nanofiber membranes loaded with 20% ZEO demonstrated complete 100% reduction in *S. aureus*, *Bacillus cereus*, and *L. monocytogenes*, with inhibition rates exceeding 60% against *E. coli* and *Salmonella typhimurium* [145]. Tayebi et al. further confirmed the effectiveness of incorporating ZEO into electrospun gelatin-based nanofibers in inhibiting *S. aureus* and *E. coli* [98]. Moreover, in another study, ZEO exhibited the inhibition of *Aspergillus niger* and *Penicillium notatum in* electrospun gelatin-based nanofibers, showing its antifungal activity [122].

In addition to the aforementioned EOs, it has also been reported that *Oliveria decumbens Vent* EO [120], *Cuminum cyminum* EO [119], cumin EO [121], ginger EO [146], galangal root oil [164], angelica EO [118,137], peppermint EO, and chamomile EO [37] can also be incorporated into electrospun gelatin-based nanofibers to impart antimicrobial activity, suitable for application in food packaging.

Some components extracted from EOs also exhibit similarly excellent antibacterial activity. For example, perillaldehyde, extracted from perilla EO, made electrospun gelatin–zein nanofiber membranes displayed effective bactericidal capability [113,114]. Betel leaf ethanol extract, rich in polyphenols and EOs, is an effective antibacterial component. Composite gelatin–chitosan nanofibers loaded with betel leaf ethanol extract were coated on PLA films, effectively inhibiting microbial spoilage in tilapia slices [108]. Also derived from plant EOs, cinnamaldehyde, thymol, carvacrol, and eugenol have been incorporated into gelatin-based electrospun nanofibers membranes, which all possessed excellent antibacterial activity [104,116,124,147,149,150,151,152].

#### 4.3.2. Integration of Antimicrobial Nanoparticles

Antimicrobial nanoparticles can be incorporated as antimicrobial agents into gelatin-based electrospun nanofiber membranes for the preparation of antimicrobial food packaging, aimed at inhibiting the growth of bacteria and fungi, thereby extending the shelf life of food.

ZnO NPs are a type of metal oxide nanoscale particle known for their excellent antimicrobial properties, often added to food packaging materials [165]. Migration studies have shown that zinc primarily transports in ion form, with negligible amounts of ZnO NPs entering the food, well below safety standards [166,167]. Ethylcellulose-gelatin electrospun nanofibers incorporated with ZnO NPs demonstrated concentration-dependent inhibitory effects against *E. coli* and *S. aureus*, with enhanced antimicrobial efficiency after UV irradiation [103]. In addition to the antimicrobial activity exhibited by ZnO NPs alone, several studies have also shown synergistic antimicrobial effects of ZnO NPs with other substances. For instance, ZnO NPs and cumin EO, when loaded together into polycaprolactone-gelatin electrospun nanofibers, exhibited synergistical antimicrobial activity against *S. aureus* [121]. Another study explored the optimal ratio of ZnO NPs and ZEO, resulting in gelatin-based electrospun nanofiber membranes with outstanding antifungal properties [122]. Moreover, ZnO NPs not only exhibited effective inhibition against *Botrytis cinerea* in gelatin–zein electrospun nanofibers but also achieved synergistic antifungal effects by altering the structure of anthocyanins [117].

In addition to ZnO NPs, there are other types of nanoparticles which have been incorporated into gelatin-based electrospun nanofibers to prepare antimicrobial food packaging. Eugenol nanoparticles can inhibit the growth of *E. coli* and *S. aureus* by releasing and diffusing through gelatin nanofibers [151]. The presence of moringa oil-loaded chitosan nanoparticles in gelatin-based electrospun nanofibers successfully inhibited the growth of *Lactobacillus plantarum* and *S. aureus* on cheese [153].

#### 4.3.3. Modification with Chitosan and Its Derivatives

Chitosan is a renewable biopolymer with inherent natural antimicrobial activity. Due to its excellent film-forming ability, biocompatibility, and biodegradability, chitosan-based films have shown immense potential in the field of food packaging [168,169]. As one of the feasible and promising methods for preparing chitosan composite fibrous materials, electrospinning can impart superior properties or achieve customized functional characteristics [170]. Given the excellent properties of gelatin and chitosan, an increasing amount of research is focusing on the development of electrospun chitosan–gelatin composite membranes for effective antimicrobial food packaging.

Tayebi et al. found that due to the presence of chitosan and *Zataria multiflora* extract, crosslinked electrospun gelatin–chitosan composite nanofiber membranes exhibited significant antimicrobial effects [98]. It not only confirmed the antimicrobial activity of chitosan but also revealed a strategy for preparing electrospun chitosan–gelatin composite nanofiber membranes with additional antimicrobial ingredients. 3-phenyllactic acid were loaded into hydrogel composed of electrospun gelatin–chitosan nanofibers or electrospun gelatin–chitosan membranes, exhibiting inhibition against foodborne pathogens [99,154]. Similarly, due to presence of nisin, PLA films coated with gelatin–chitosan nanofibers demonstrated excellent antimicrobial activity [107]. Furthermore, the co-loading of chitosan with curcumin or ε-PL into electrospun gelatin nanofiber membranes demonstrated a synergistic antimicrobial effect [109,155].

Chitooligosaccharide, a derivative of chitosan obtained through chemical or enzymatic hydrolysis, also possesses excellent antibacterial properties [171,172]. The inclusion of chitooligosaccharide in fish skin gelatin electrospun nanofiber hydrogels has demonstrated outstanding antibacterial capability, effectively extending the shelf life of crucian carp [132]. Furthermore, chitosan oligosaccharides can synergistically enhance antimicrobial efficacy when combined with chitosan. Gulzar et al. prepared electrospun gelatin–chitosan nanofibers loaded with tannic acid and chitooligosaccharides onto PLA films; the composite films exhibited increased antibacterial activity against both Gram-positive and Gram-negative bacteria [156].

#### 4.3.4. Incorporation of Other Antibacterial Agents

In addition to the aforementioned types of antimicrobial agents, various other antimicrobial agents have also been incorporated into gelatin-based electrospun nanofiber membranes to prepare antimicrobial food packaging.

Plant extracts offer promising options. Fucoxanthin, a natural pigment widely present in various algae, when incorporated into gelatin-based electrospun nanofibers, imparted a certain degree of antimicrobial ability to the membrane [157]. Similarly, sage extract incorporated into gelatin–zein composites either through electrospinning or solvent casting, gelatin–zein composites demonstrated significant antimicrobial activity [158]. Moreover, propolis [134] and resveratrol [38] can also be incorporated into electrospun gelatin-based nanofibers, respectively, imparting antimicrobial activity.

Chemically synthesized substances are also a suitable addition for electrospun gelatin-based membranes, applying to antibacterial food packaging. The electrospun gelatin-based membranes doped with low concentrations of lauroyl arginine ethyl possessed antibacterial activity, which could be applied to extend the shelf life of yellow croaker filets [159]. An electrospun gelatin mat loaded with allyl isothiocyanate supporting a pressure-sensitive adhesive demonstrated significant inhibition of *S. aureus* and *E. coli*, and extended the shelf life of cheese by 4 weeks [160]. Furthermore, successful encapsulation of glycerol monolaurate microemulsion [138] and butylated hydroxyanisole, respectively, in gelatin-based electrospun nanofibers enabled the effective inhibition of *S. aureus*, *Rhizopus* sp., *Mucor* sp., *Aspergillus* sp., and *Penicillium* sp.

In particular, the incorporation of certain probiotics in gelatin-based electrospun nanofibers has also been demonstrated antibacterial activities. Ghalehjooghi et al. developed an active packaging nanofiber mat based on gelatin-sodium alginate containing probiotic microorganisms, which could delay the growth of foodborne pathogens [126]. Derived from microbial strains, ε-PL is a cationic polypeptide known for its broad-spectrum antimicrobial activity [173]. Several studies have incorporated ε-PL into electrospun gelatin-based membranes, which exhibited significant inhibitory activity against many kinds of bacteria [105,109,115,148,162].

In conclusion, the development of gelatin-based electrospun antimicrobial packaging focuses on four strategic approaches: (1) the encapsulation of plant-derived essential oils (EOs), (2) the integration of antimicrobial nanoparticles (e.g., ZnO, silver), (3) modification with chitosan and its derivatives, and (4) the incorporation of bioactive compounds (e.g., ε-polylysine, enzymes). These agents are embedded within gelatin nanofibers via electrospinning, leveraging their inherent antimicrobial properties to inhibit microbial growth through mechanisms such as membrane disruption, enzyme inactivation, and sustained ion release. EOs provide broad-spectrum activity with natural safety, while chitosan enhances both antibacterial efficacy and material stability. Nanoparticles offer prolonged antimicrobial action due to controlled release kinetics, and synergistic combinations (e.g., EO-nanoparticle hybrids) further amplify performance. The versatility of electrospinning enables the precise tuning of agent distribution and fiber morphology, ensuring optimal functionality without compromising gelatin’s biodegradability. Future research should prioritize optimizing agent compatibility, assessing long-term safety in food contact applications. This multi-strategy framework positions gelatin-based electrospun materials as adaptable solutions for next-generation antimicrobial food packaging.

### 4.4. Antioxidant Functionalization

Oxidation reactions are not confined to the human body, they also transpire in food items, resulting not only in a decline in their nutritional value but also potentially leading to health issues due to oxidative spoilage [174,175]. To address the problem, strategies typically involve the direct addition of antioxidants to the food or improvements in food packaging. However, adding antioxidants directly to food may affect its appearance and taste, and the excessive use of antioxidants in food may have adverse effects on human health. Therefore, the development of antioxidant food packaging represents a superior approach to solving the issue of food oxidation.

**Figure 7:**
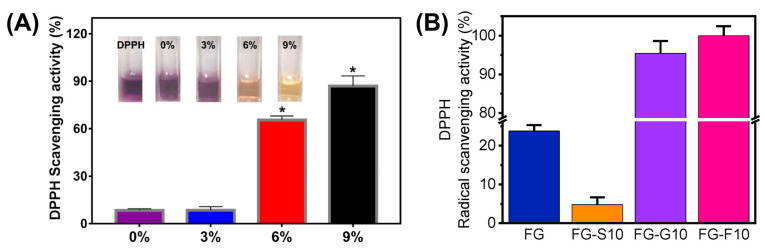
Some representative studies of electrospun gelatin-based nanofibers incorporated with antioxidant agents. (**A**) Antioxidant activity of electrospun gelatin nanofibers containing different content angelica EO. Reprinted with permission from reference [118]. Copyright 2020 Multidisciplinary Digital Publishing Institute. * *p* < 0.05 versus the control group. (**B**) Antioxidant activity of electrospun crosslinked fish gelatin nanofibers containing sucrose, glucose, or fructose. FG = fish gelatin, S = sucrose, G = glucose, F = fructose, 10 = 10% (w/w, based on the gelatin weight). Reprinted with permission from reference [177]. Copyright 2021 Elsevier.

Electrospinning technology offers distinct advantages in fiber structure and functionality, allowing efficient encapsulation of antioxidants to functionalize nanofibers [13,52]. Despite exhibiting certain antioxidant activity due to the presence of peptides, gelatin alone may not sufficiently meet the requirements for antioxidant packaging materials. In recent years, gelatin has frequently been employed as a substrate material in electrospinning to facilitate the incorporation of active agents for the preparation of antioxidant food packaging (Figure 7).

#### 4.4.1. Encapsulation of Phenolic Compounds

Phenolic compounds are characterized by the presence of phenol groups. Due to the high reactivity of the hydroxyl group and their ability to scavenge free radicals, phenolic compounds are renowned for their antioxidant properties, which are excellent candidates as active agents integrated into gelatin-based electrospun nanofibers for the development of antioxidant food packaging. Resveratrol, a non-flavonoid polyphenolic compound, predominantly exists in natural plants. The loading of resveratrol has been demonstrated to significantly enhance the antioxidant activity of electrospun gelatin–zein nanofiber membranes [38]. Curcumin, a natural polyphenolic compound, when added to electrospun gelatin–chitosan nanofiber membranes, can enhance the antioxidant capability of the nanofiber membranes [155]. Chlorogenic acid, a polyphenolic secondary metabolite widely found in plants, derives its antioxidant activity from its ortho-dihydroxy phenolic structure. Chlorogenic acid was incorporated as a natural antioxidant into electrospun fish gelatin nanofibers by Estevez-Areco et al. [133], and they found that the introduction of crosslinking agents such as citric acid or fructose can indirectly enhance the antioxidant effect by increasing the water resistance of the nanofiber membrane, thereby extending the release of antioxidants. Similarly, due to the presence of phenols, successful loading of sage extract, betel leaf ethanolic extract, propolis, pine honey gallic acid, and proanthocyanidin have also been reported to enhance the antioxidant activity of gelatin-based electrospun nanofiber membranes [108,115,117,125,134,158].

Owing to the rich content of phenolic compounds, plant EOs typically exhibit remarkable antioxidant activity. EOs extracted from aromatic plants are commonly utilized as natural antioxidants in the development of antioxidant food packaging [176]. It has been reported that chamomile EO, peppermint EO, angelica EO, ZEO, and ginger EO have also been added as natural antioxidants to electrospun gelatin-based nanofibers [37,118,122,144,146].

Another strategy is to directly incorporate the specific phenolic components extracted from EOs as antioxidants into gelatin-based electrospun nanofibers. Carvacrol, a monoterpenoid phenol compound widely found in the EOs of plants such as thyme and oregano, is highly regarded in the field of food packaging due to its excellent antioxidant and antibacterial properties. When carvacrol and γ-cyclodextrin were co-doped into gelatin-based electrospun nanofibers, the membrane exhibited outstanding antioxidant activity due to the chain-breaking activity of phenolic components [150]. Another report also indicated that carvacrol was able to delay lipid oxidation and enhance the antioxidant activity of gelatin-based electrospun membranes [149]. Similarly, thymol, eugenol, and perillaldehyde have also been incorporated into electrospun gelatin-based nanofibers, enhancing the antioxidant activity of nanofiber membranes [113,114,116,147,151].

#### 4.4.2. Integration of Other Antioxidant Components

In addition to the phenolic compounds mentioned above, several other components have also been studied to enhance the antioxidant performance of gelatin-based electrospun nanofibers. For instance, due to the rich content of amino acid residues, *Spirulina* protein was reported to enhance the antioxidant activity of electrospun gelatin nanofibers [139]. The gelatin-based electrospun membrane doped with fucoxanthin was also reported to exhibit excellent antioxidant properties, attributed to the carotenoid structure of fucoxanthin [157]. With an aldehyde group and double bond, cinnamaldehyde can also effectively prevent lipid oxidation and scavenge free radicals. Its significant antioxidant activity has been validated in gelatin–zein electrospun nanofiber membranes by Wu et al. [104]. By embedding chitooligosaccharides, gelatin-based electrospun nanofibers exhibited excellent antioxidant activity, due to the metal ion chelation efficiency of chitooligosaccharide’s amino groups [132,156]. Interestingly, Kwak et al. found that the incorporation of sucrose, glucose, or fructose into electrospun gelatin-based nanofibers could also enhance the antioxidant properties due to the production of Maillard reactants in a mild heat treatment crosslinking process [177].

The design of gelatin-based electrospun antioxidant packaging centers on two principal strategies: (1) the encapsulation of phenolic compounds (e.g., plant extracts, polyphenols) and (2) the integration of other antioxidant agents (e.g., vitamins, carotenoids, enzymes). These components are embedded within gelatin nanofibers via electrospinning, leveraging their radical-scavenging and chain-breaking capabilities to mitigate oxidative degradation in packaged foods. While gelatin inherently exhibits mild antioxidant activity due to its peptide content, synergistic combinations with exogenous antioxidants significantly enhance performance. Phenolic compounds, such as those derived from botanical sources, provide potent hydrogen-donating activity, whereas non-phenolic agents (e.g., tocopherols) inhibit lipid peroxidation through electron transfer mechanisms. Electrospinning ensures uniform antioxidant distribution and controlled release kinetics, minimizing direct food additive interactions while preserving sensory qualities. This approach circumvents the drawbacks of conventional antioxidant addition, such as taste alteration and potential health risks from excessive dosing. Future advancements should focus on optimizing antioxidant–gelatin compatibility, evaluating long-term stability under storage conditions, and developing eco-friendly extraction methods for bioactive agents to align with sustainable packaging paradigms.

### 4.5. Functionalization for Intelligent Food Packaging

Intelligent food packaging is often defined as a system capable of monitoring the condition of the packaged food or its surrounding environment while conveying relevant information about food freshness to consumers, providing a basis for its assessment [178]. Drawing upon referenced reviews and the integration of definitions by multiple researchers, it is summarized that intelligent packaging generally encompasses six main functions: monitoring, detecting, sensing, recording, tracking, and communicating [179]. Various sensing modalities have been employed, including time, temperature, humidity, oxygen, pH, chemical composition, or microbial contamination [180].

To achieve the functionalities of monitoring, detection, and sensing, intelligent packaging requires a suitable “platform”. Electrospinning technology is a potential production technique advantageous in manufacturing such a platform. The high surface area-to-volume ratio, absorbance, porosity, and small pore size of electrospun nanofiber membranes are conducive to developing intelligent packaging, enabling real-time monitoring, rapid response, and visualization of food freshness [181].

**Figure 8:**
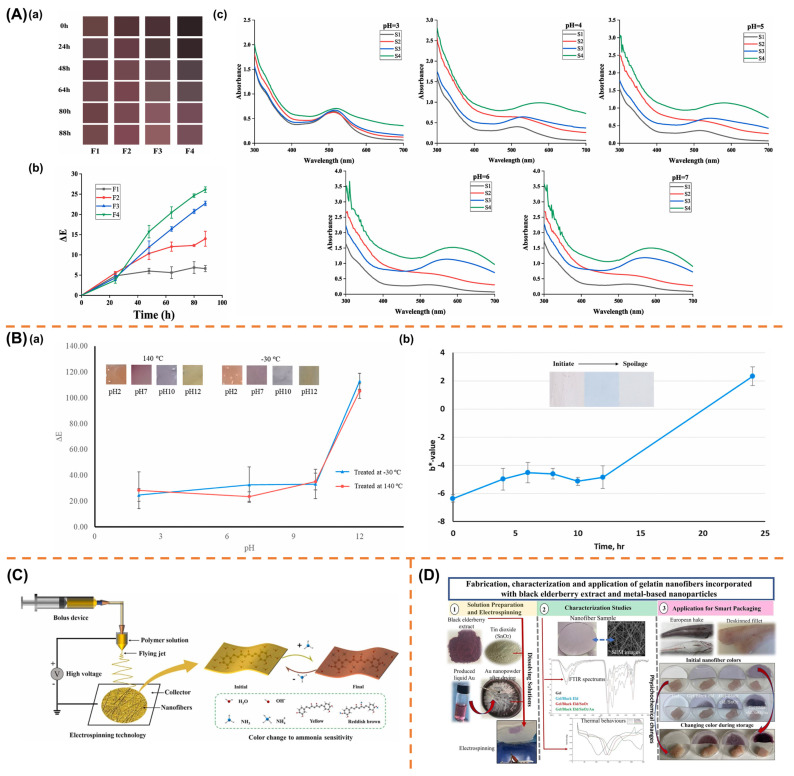
Electrospun gelatin nanofiber membranes for intelligent food packaging. (**A**) pH indication effect (**a**–**c**) of electrospun zein–gelatin–Fe^2+^–blueberry anthocyanins nanofiber indicator film. Reprinted with permission from reference [182]. Copyright 2022 Elsevier. (**B**) Stability (**a**) and color change (**b**) of electrospun pH-responsive gelatin-based nanofibers loaded with red radish anthocyanin extract. Reprinted with permission from reference [183]. Copyright 2023 Elsevier. (**C**) Preparation and mechanism for color change in electrospun gelatin–chitosan nanofibers loaded with curcumin. Reprinted with permission from reference [155]. Copyright 2023 KeAi Publishing. (**D**) Schematic of electrospun gelatin nanofibers incorporated with black elderberry extract, Au nanoparticles, and SnO_2_. Reprinted with permission from reference [184]. Copyright 2024 Elsevier.

The construction of the “platform” typically involves the incorporation of suitable components into gelatin-based electrospun nanofibers as sensing elements (Figure 8). For dairy products, a gelatin-based nanofiber colorimetric indicator membrane capable of monitoring the freshness of milk was prepared [182]. The interaction among gelatin, Fe^2+^, and blueberry anthocyanins enhanced the sensitivity of the nanofiber indicator membrane to pH color response, and visual color changes could intuitively indicate the acidity of milk, reflecting its freshness. A stable electrospun gelatin nanofiber membrane incorporating anthocyanin extracts from red carrots has been reported, and demonstrated the capability to exhibit significant color changes reliably through respond pH, reflecting the freshness level of the meat [183]. Due to the successful loading of curcumin, electrospun gelatin–chitosan nanofiber membranes exhibited high sensitivity in the colorimetric behavior towards ammonia, making them suitable for monitoring the freshness of protein-rich animal-derived foodstuffs [155]. Owing to the interaction between newly formed Au-Sn metallic nanostructures in the nanofiber membrane and the volatile compounds generated during fish oxidation, electrospun gelatin-based nanofiber doped with black elderberry extract, Au nanoparticles, and SnO_2_ could exhibit rapid color response to the spoilage of cod fish slices, showing its potential in monitoring the quality of seafood products [184].

To sum up, gelatin-based electrospun nanofibers are emerging as versatile platforms for intelligent packaging systems, primarily leveraging their high surface area, porosity, and tunable responsiveness to environmental stimuli. Current applications predominantly focus on pH-sensitive indicators, such as anthocyanin- or curcumin-loaded nanofibers, which enable visual freshness monitoring in dairy, meat, and seafood products through colorimetric changes triggered by pH shifts or spoilage metabolites (e.g., ammonia, volatile compounds). While these systems demonstrate gelatin’s compatibility with bio-based sensors, existing studies remain limited in scope, with minimal exploration of other sensing modalities (e.g., gas, temperature, microbial detection). The integration of advanced components—such as metallic nanoparticles (Au, SnO_2_) and redox-active extracts—hints at broader potential for multi-parametric monitoring. However, the field currently lacks standardized protocols for sensor sensitivity optimization and long-term stability under real-world conditions. Future research should prioritize diversifying sensing mechanisms (e.g., enzyme-based or gas-responsive systems), enhancing signal accuracy through nanostructure engineering, and addressing scalability challenges in electrospinning production. Bridging these gaps will unlock gelatin’s untapped potential in next-generation intelligent packaging, aligning with demands for real-time food quality tracking and reduced waste in sustainable supply chains.

## 5. Summary and Prospect

With the improvement of living standards, people are increasingly prioritizing food safety and environmental protection. As frontline defenses for safeguarding food safety, food packaging needs to evolve to meet new demands such as safety, environmental friendliness, and functionality. Electrospun gelatin-based nanofiber membranes have shown significant potential in this new trend. Compared to many non-degradable traditional packaging materials, the naturally sourced gelatin presents significant advantages as environmentally friendly food packaging. Electrospinning not only offers high encapsulation efficiency and relaxed operating conditions but also produces nanofibers with larger surface area, higher porosity, and greater surface modifiability, which can be customized according to requirements, thereby facilitating the development of functional food packaging. In recent years, electrospun gelatin-based nanofiber membranes have shown great potential as functional food packaging. For instance, the incorporation of active substances rich in hydrophobic groups or combining electrospinning with other technologies to produce composite films, multilayer films, or crosslinked films can improve the water barrier properties of electrospun gelatin-based nanofiber membranes, thus extending the shelf life of food. Edible food packaging derived from environmental concepts can be achieved through electrospinning gelatin-based nanofibers co-doped with natural active substances. For the development of antibacterial food packaging and antioxidant food packaging, various antibacterial agents or antioxidants can also be easily incorporated into electrospun gelatin-based nanofibers. Furthermore, although current research is still in its initial stages, modified electrospun gelatin-based nanofibers continue to demonstrate their potential applications as smart food packaging materials.

However, there are still some challenges and shortcomings at present. Firstly, there is still much untapped potential for existing modified functional electrospun gelatin-based nanofibers. The functionality of nanofiber membranes can be expanded to incorporate multiple properties, such as combined antibacterial, antioxidant, and moisture retention functions. To address this, future research should focus on optimizing the formulation and electrospinning parameters to simultaneously achieve multiple functions with minimal processing complexity. Furthermore, the development of electrospun gelatin-based nanofiber membranes with tailored functionalities should explore incorporating various biomaterials and natural polymers to achieve multifunctionality in a cost-effective manner. Secondly, currently developed intelligent packaging response mechanisms based on electrospun gelatin-based nanofibers for monitoring food quality are limited. However, food quality is influenced by multiple factors, and relying on a single response mechanism may limit the effectiveness of the packaging in real-world applications. To improve the functionality and adaptability of intelligent food packaging, further development of electrospun gelatin-based nanofiber membranes should focus on incorporating multiple response mechanisms into a single system, with future exploration directed toward such integrations or more suitable packaging solutions. This is promising and feasible for electrospinning gelatin-based nanofibers. There have been studies that successfully prepared moisture and pH dual responsive smart nanofiber antibacterial packaging [185] as well as enzyme and relative humidity responsive antibacterial nanofiber active food packaging [186]. Additionally, employing multifunctional nanofibers that can incorporate a combination of chemical, biological, and physical stimuli would enhance the system’s sensitivity and specificity.

Moreover, the transition of laboratory achievements to large-scale industrial production remains a critical challenge. According to a market analysis report by Grand View Research on food packaging [187], the global food packaging market reached USD 400.29 billion in 2024, with a projected compound annual growth rate (CAGR) of 5.9% from 2025 to 2030, indicating a period of active industry development. The prevailing sustainability paradigm has driven both industries and regulatory bodies to promote innovations in biodegradable and eco-friendly materials. For instance, U.S.-based Greif introduced Coated Recycled Paperboard specifically designed for food packaging applications. Amcor collaborated with Avantium NV (the FDCA Flagship Plant in Delfzijl, The Netherlands) to explore renewable and circular polymer materials while securing commitments for future industrial-scale production capacity. Additionally, Amcor partnered with Kolon Industries to develop commercially recyclable sustainable packaging solutions. In alignment with environmental objectives, the European Commission proposed updated packaging regulations in 2022 to reduce packaging waste and enhance reuse/refill systems. Nevertheless, industrializing electrospun nanofiber membranes, including gelatin-based ones, requires addressing critical challenges including production capacity and cost-effectiveness. Most laboratory-scale setups employ needle-based electrospinning equipment, which delivers adequate productivity for research purposes but proves insufficient for industrial demands [188]. While some enterprises have advanced industrial applications of electrospinning technology—notably China’s Foshan Weima Technology Company (Foshan, China) in waterproof breathable membranes, medical films, and filtration membranes—current progress remains limited. Innovative approaches like the polymer melt differential electrospinning method reported by Chen et al. [189] demonstrate potential for scaling up nanofiber production. However, electrospinning technology has yet to mature into a widely adopted industrial process. Future research should prioritize bridging the gap between laboratory innovations and industrial implementation. Collaborative efforts with industry partners are essential to address practical challenges in production multifunctionality, cost optimization, and technology standardization. Such partnerships will accelerate the translation of electrospinning advancements into commercially viable solutions for real-world applications.

Finally, the development of functional food packaging based on electrospinning technology is still in its early stages, facing more exploration and challenges in the future, which also represents the enormous potential of electrospinning functional food packaging. In the face of more complex problems, it is necessary to strengthen interdisciplinary cooperation to jointly promote the development of the field.

## Figures and Tables

**Table 1:** Food packaging prepared by different electrospun techniques.

Electrospinning Technique	Material	Collector	Reference
Single-Nozzle Electrospinning	Cocktail and zein	Aluminum foil	[58]
	Polylactic acid (PLA) and tea polyphenol	Aluminum foil	[59]
	Seed meal protein and pullulan	Drum collector	[60]
Coaxial Electrospinning	Cinnamaldehyde, tea polyphenols, and polylactic acid (PLA)	Drum collector	[61]
	Ethylene vinyl alcohol copolymer and thymol	metallic collector	[62]
	Polyethylene oxide (PEO), zein, resveratrol, and silver	metallic collector	[63]
	Thymol, genipin, polyethylene oxide (PEO), and chitosan (CS)	Aluminum foil	[64]
Emulsion Electrospinning	Basil oil, zein, and alginate	Baking paper collector	[65]
	β-carotene, soy protein, and polyvinyl alcohol (PVA)	Drum collector	[66]

## Data Availability

No data were used for the research described in the article.
